# Is the postoperative discomfort after connective tissue harvesting from the palate influenced by the use of a bipolar coagulator? A randomized controlled trial

**DOI:** 10.1002/cre2.441

**Published:** 2021-05-13

**Authors:** Federico Tirone, Stefano Salzano, Paola Panuello, Laura Pozzatti, Donata Rodi

**Affiliations:** ^1^ Clinica Odontoiatrica Salzano Tirone, Private Practice Cuneo Italy; ^2^ Freelance Statistician Ferrara Italy

**Keywords:** bipolar coagulator, complications, connective tissue graft, pain, palatal tissue harvesting

## Abstract

**Objectives:**

This study aimed to determine the effect of the use of a bipolar coagulator on postoperative pain and complications when used during connective tissue harvesting from the palate.

**Material and methods:**

A randomized controlled clinical trial was conducted with 57 sequential patients requiring a connective tissue graft for periodontal or implant surgery. All samples were harvested superficially and de‐epithelized outside the mouth. The patients were randomly allocated to two groups: in one group, the bipolar coagulator was used before suturing to control bleeding, and in the other group, the coagulator was not used. The surgeon was unaware of the randomization until the end of the harvesting phase. Self‐reported maximum pain, number of painkillers used, bleeding events, emergency visits at the clinic were recorded 7 days after surgery.

**Results:**

Fifty patients were randomized and treated (seven were excluded for different reasons). The mean harvested area was 75.24 mm^2^ (SD, 33.96), and the mean thickness of the samples was 2.47 mm (SD, 0.75). The mean self‐reported pain value on the visual analog scale was 3.37 (SD, 2.30), and the mean number of pain medications used was 7.1 (SD, 6.60). Seven patients made an emergency visit each, and 17 delayed bleeding events were reported by 15 patients. No statistically significant differences were reported in postoperative pain, postoperative bleeding, and emergency visit to the clinic between the groups that did and did not use the bipolar coagulator. When smoking habits were taken into consideration, the number of pain medications was higher among male smokers and older smokers than among male non‐smokers and younger smokers. This study was not able to find a relationship between harvested sample dimension or thickness and postoperative discomfort.

**Conclusions:**

The bipolar coagulator can be used during connective tissue harvesting from the palate to control bleeding without influencing postoperative pain.

## 1 INTRODUCTION

Connective tissue (CT) harvesting from the palate has been widely used and described in the past as a fundamental step in bilaminar mucogingival surgery (Langer & Langer, 1985; Tonetti & Jepsen, 2014). Improved root coverage has been shown both in localized gingival recessions (Cairo et al., 2014; Cortellini et al., 2009) and multiple recessions (Graziani et al., 2014; Pini‐Prato et al., 2010; Zucchelli, Mounssif, Mazzotti, Stefanini, et al., 2014) when a connective tissue graft (CTG) was added to the coronally advanced flap. Furthermore, recent data show that it is possible to obtain complete root coverage even if some interdental attachment has been lost (Cairo et al., 2012). Considering the ever‐increasing use of dental implants in the last few decades, there is a new interest in the use of CTGs in the field of implant surgery to improve ridge volume even when shallow buccal dehiscence is present around an implant (Esposito et al., 2012; Stefanini et al., 2016; Tirone & Salzano, 2018). As a consequence, autologous CTG harvest from the palate is a current topic. However, to date, no soft tissue substitutes have demonstrated the same predictability as that of autologous CTGs in increasing soft tissue thickness or reducing the probability of gingival recession (Jepsen et al., 2013; Schmitt et al., 2019).

The first harvesting technique, namely the trapdoor technique, was described by Edel in 1974 (Edel, 1974). Later, several modifications of the subepithelial harvesting technique were described (Broome & Taggart Jr., 1976; Bruno, 1994; Del Pizzo et al., 2002; Hürzeler & Weng, 1999; Langer & Calagna, 1980; Langer & Langer, 1985). Recently, a de‐epithelized gingival graft‐harvesting technique requiring de‐epithelization outside the mouth and second‐intention healing of the donor site was described (Zucchelli et al., 2003). This technique seems to yield better aesthetic outcomes and higher‐quality connective tissue, without adipose and glandular tissue, than other techniques. Two randomized clinical trials (RCTs) found a statistically significant difference in postoperative pain between the free‐gingival graft harvesting procedure (more painful) and sub‐epithelial CTG harvesting technique (Del Pizzo et al., 2002; Wessel & Tatakis, 2008), but an RCT from Zucchelli and coworkers using a large sample and the previously quoted technique (Zucchelli et al., 2003) found no difference between sub‐epithelial and epithelia‐connective harvesting, in terms of postoperative pain and discomfort (Zucchelli et al., 2010).

Bleeding is a potential peri‐ and postoperative complication of palatal harvesting, and a bipolar coagulator is useful in achieving hemostasis during surgery. The effects of using a bipolar coagulator in palatal harvesting procedures have never been described, although this particular instrument is widely used in other surgical fields (Advincula & Wang, 2008; Cannizzaro et al., 2016; Janssen et al., 2012; Tirelli et al., 2018).

The aim of this study was to evaluate the effect of using a bipolar coagulator during CTG palatal harvesting, via the epithelia‐connective technique, on the patient's healing experience and on the occurrence of post‐surgical complications. Considering the lack of previous studies on this topic, a hypothesis was not proposed in this study.

## 2 MATERIAL AND METHODS

This trial was reported according to the Consolidated Standard of Reporting Trials Statement (http://www.consort-statement.org/). All the procedures were approved by the institutional review board of Salzano Tirone dental Clinic and were carried out in compliance with the ethical principles for medical research involving human participants, as stated in the Declaration of Helsinki. The present study was designed as an RCT and was conducted in private practices, run by a dentist who has been practicing oral surgery for more than 15 years (FT), located in northern Italy.

All consecutive healthy patients who needed CTG for periodontal or implant surgery were considered eligible. Patients were not recruited to the study if any of the following exclusion criteria were present: clinically significant medical history (e.g., systemic infective disease, heart and vascular disease, liver disease, hematological disease, coagulation deficiency, diabetes, and neoplastic disease), immunosuppressed or immunocompromised state, those who received radiotherapy to the head and neck area, those requiring bone augmentation procedures concomitant with implant placement, allergic to some of the drugs involved in the protocol, receiving antibiotic treatment for other reasons, treated or receiving treatment with intravenous amino‐bisphosphonates, affected by untreated periodontitis, full mouth plaque score and full mouth bleeding score >15, pregnant or lactating females, and age under 18 years.

All patients were informed about the surgical procedures, postoperative follow‐ups, and potential complications, and informed consent was obtained before surgery. Data on smoking habits were acquired.

One hour prior to surgery, the patients took a single antibiotic dose, consisting of 2 g of oral amoxicillin (two tablets of 1 g) and 10 drops of ketorolac. The mouth was rinsed for 1 min immediately prior to implant placement with 0.2% chlorhexidine gluconate mouthwash.

CT harvesting was performed following the FGG harvesting approach as previously described by Zucchelli et al. (2003). First, a horizontal split thickness incision parallel to the occlusal plane was made in the palatal zone between the second molar and the second premolar, approximately 4 mm apical to the palatal gingival margin of the maxillary posterior teeth. The length was 1 mm greater than the CTG length needed. Then, two 5‐mm‐long vertical incisions were made, and a homogeneous‐thickness sample was dissected and finally detached with a second apical horizontal incision. The periosteum and the deep part of the submucosal tissue were left in place.

The patients were randomly allocated to one of two groups using a closed envelope to be opened after CT harvesting: Group A: bipolar coagulator was not used, and bleeding was managed with x sutures (5–0 polyglycolic acid; Omnia®, Fidenza, Italy) and by fixing a collagen sheet over the donor site (Condress®, Smith & Nephew Medical®, Hull, England); Group B: bipolar coagulator (Diatermo MB 120D; Gima®, Gessate, Italy) was used in the inner part of the wound boundaries (power 40 W) until hemostasis was obtained before suturing; later, the same suturing technique was performed fixing a collagen sheet in the same way. In both groups, a wet gauze was pushed against the donor site for a few minutes during extraoral de‐epithelization of the graft.

The dimensions of the sample were based on the requirement for recipient site. Before de‐epithelization, the sample was measured in millimeters with a periodontal probe, and the length, height, and width were recorded. The presence or absence of adipose yellow tissue was recorded as follows: no fat tissue, less than 50% of the inner surface of the sample covered by fat tissue, and more than 50% of the inner surface of the sample covered by fat tissue.

Postoperative instructions and ice packs were provided to the patient immediately after surgery. The patients were instructed to rinse with 0.12% chlorhexidine gluconate twice daily for 15 days after surgery and to refrain from brushing the area of surgery for 2 weeks and were prescribed amoxicillin 1 g, twice a day, for 6 days and ketorolac for 3 days, if needed (10 drops, corresponding to 10 mg, when needed with at least 4 hours between each use; maximum dose: 40 drops per day).

The patients were given a diary to record the pain medication used and the occurrence of bleeding; the diary had to be signed and delivered at suture removal, 1 week after the surgical procedure. Before suture removal, the patients were asked to fill a visual analog scale (VAS) from 0 (no pain) to 10 (worst pain) to determine the maximum pain experienced during the first healing week. The outcome data were recorded by a blinded assessor (SS).

Follow‐up visits were conducted at 1 week (suture removal), 2 weeks, 1 month, and 2 months after surgery.

The primary outcome measures were as follows:

- Maximum pain experienced by the patient along the first week of healing, as recoded on the VAS 7 days after surgery.
- Number of pain medications used, excluding the “mandatory” one before surgery.

The secondary outcome measures were as follows:

- Reported delayed bleeding events from the donor site, described as prolonged hemorrhage after discharge, recorded as “yes” or “no.”
- Emergency access to dental practice.

The sample size was difficult to estimate because of the lack of validated data on implant patients treated with these regimens. Therefore, to perform the calculation, we hypothesized a difference of two points in the VAS score between the groups as clinically important, with a power of 0.80 and *α* = 0.05. An effect size of 0.87 was obtained using Cohen's table for two independent groups. As per the G‐Power software, the number of subjects required per group was 23.

A computer‐generated randomization list comprising 50 subjects was created. Only one external investigator, not involved in the study, was aware of the sequence and had access to the file. The randomized codes were enclosed in sequentially sealed envelopes, opened at the end of the CT withdrawal before suturing.

For data analysis, the following codes were defined: treatment (0, control; 1, treated), adipose tissue (0, no fat; 1, fat <50%; 2, fat ≥50%), smoke more than 5 cigarettes for day (0, no; 1, yes), emergency visit (0, no; 1, yes), and bleeding (0, no; 1, yes). Considering the results of the assumptions for the parametricity of data, non‐parametric tests were performed. To analyze the effect of bipolar coagulation treatment and smoking habit on VAS and the number of pain medications used, Mann–Whitney *U* test was used. To analyze the effect of adipose tissue on VAS and number of pain medications used, Kruskal–Wallis test was applied, followed by Dunn's post‐hoc test. Then, the sample was stratified by gender (1, male; 2, female) and by age (class 1, 18–40 years; class 2, 41–49 years; class 3, ≥50 years). Binomial logistic regression was used to analyze the effect of coagulation treatment, smoking, fat, area, and thickness of the sample on emergency visits and bleeding.

All analyses were conducted using GraphPad Prism (Version 6.0) and SPSS (Version 22.0).

## 3 RESULTS

**FIGURE 1:**
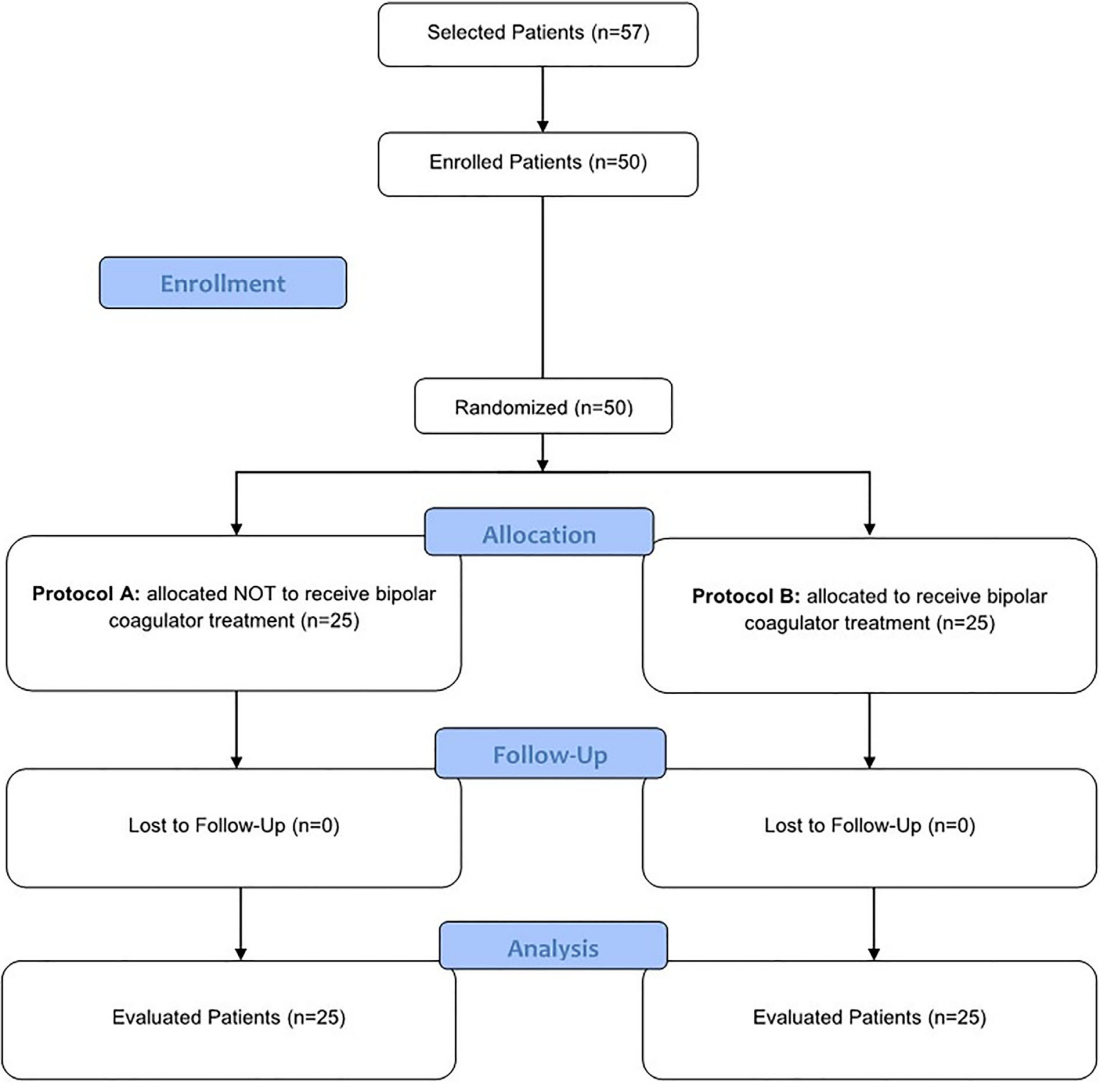
Flow chart – CONSORT

Initially, 57 patients needing CTG for either periodontal or implant surgery were screened during the recruitment period. Six of these patients were not included (five had clinically significant medical history; one reported ketorolac allergy). One patient refused to participate in this study. Figure 1 shows the flow diagram of patient enrolment and selection.

Finally, 50 patients were randomized and treated. The follow‐up period was the time between implant placement and 3 months after the surgical procedure. No deviations from the operative protocol occurred.

The patient and treatment characteristics are described in Table 1. Twenty CTGs were used for mucogingival surgery and 30, for implant surgery; the bipolar coagulator was used in 25 cases, while it was not used in 25 patients. No patient dropped out during the 3‐month follow‐up period.

**TABLE 1:** Patient and treatment characteristics

	GROUP A°	GROUP B°
Number of patients	25	25
Number of patients with available data	25	25
Age (SD^a^)	45.4 (12.4)	46.77 (12.40)
Male:Female	10:15	8:17
Smokers	3 (12%)	4 (16%)
Implant purpose	18	12
Periodontal purpose	7	13

*Note*: Group A = bipolar coagulator was not used; Group B = bipolar coagulator was used before suturing.

^a^ SD, standard deviation.

The characteristics of the harvested samples are presented in Table 2. The mean harvested area was 75.235 (SD, 33.96), and the mean thickness of the samples was 2.47 (SD, 0.75). The results are summarized in Table 3. The mean self‐reported pain VAS value was 3.37 (SD, 2.30), and the mean number of pain medications used was 7.1 (SD, 6.60). Seven emergency visits were made by seven patients, and 17 bleeding events were reported by 15 patients.

**TABLE 2:** Characteristics of the harvested samples

	GROUP A°	GROUP B°
Patients	25	25
Harvested sample length (SD^b^)	5.1 (0.9)	5.0 (1.0)
Harvested sample width (SD^b^)	16.0 (6.3)	13.5 (3.4)
Harvested sample thickness (SD^b^)	2.6 (0.9)	2.4 (0.6)
Fat tissue class 0^a^	12	10
Fat tissue class 1^a^	6	7
Fat tissue class 2^a^	7	8

*Note*: Group A = bipolar coagulator was not used; Group B = bipolar coagulator was used before suturing.

^a^ Class 0 = no adipose (yellow) tissue in the inner surface of the harvested tissue; class 1 = <50% of the inner surface of the tissue covered by adipose tissue; class 2 = ≥50% of the inner surface of the tissue covered by adipose tissue.

^b^ SD, standard deviation.

**TABLE 3:** Odds ratios for emergency visit

	Wald *t* test	*p*‐value	Odds ratio	95% CI°
Bipolar (yes vs. no)	1.346	0.246	2.954	0.474–18.425
Area	0.041	0.840	1.003	0.978–1.027
Thickness	0.657	0.418	0.579	0.154–2.173
Fat (1 vs. 0)	2.409	0.121	6.75	0.605–75.270
Fat (2 vs. 0)	0.077	0.782	1.500	0.085–26.361

*Note*: Logistic binomial regression test for bipolar, area, and thickness variables on emergency visit on global and adipose‐tissue stratified data. °CI, confidence interval.

**FIGURE 2:**
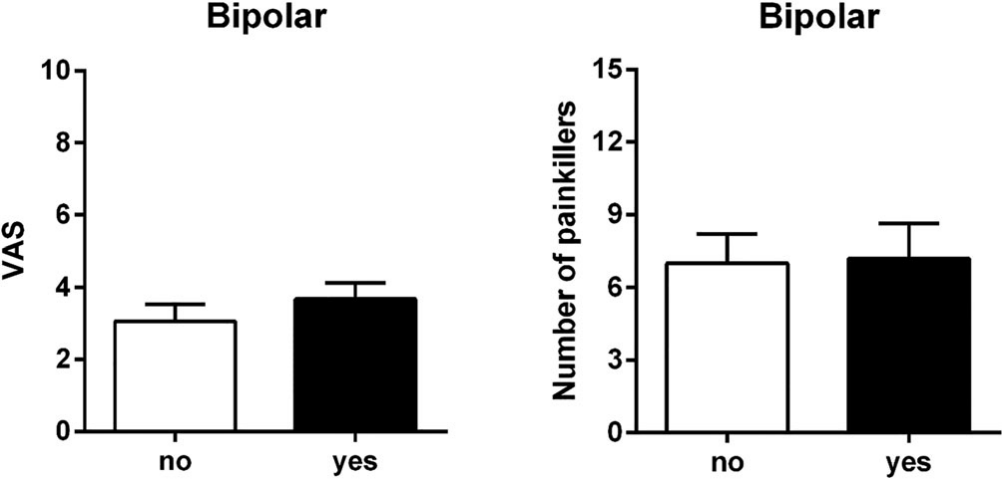
Effect of coagulation on VAS and number of pain medications taken. Coagulation treatment group (black bar), control group (white bar). *P* = 0.289 (VAS); *P* = 0.768 (Painkillers), Mann–Whitney *U* test

No statistically significant differences were found with and without the use of the bipolar coagulator, as per the 7‐day VAS outcome (*U* = 258.500; *P* = 0.289) or the number of pain medications (*U* = 297.500; *P* = 0.768) (Figure 2). No statistically significant differences in maximum pain (VAS) were found when considering smoking habits (*U* = 124.000; *P* = 0.453), sample area (chi‐square = 0.654; *P* = 0.721), sample thickness (chi‐square = 5.773; *P* = 0.217), and the presence of yellow adipose tissue (chi‐square = 3.701; *P* = 0.157).

Similar results were found regarding the number of painkiller medications used, considering smoking habits (*U* = 105.500; *P* = 0.202), sample area (chi‐square = 2.407; *P* = 0.300), sample thickness (chi‐square = 2.130; *P* = 0.712), and the presence of yellow adipose tissue (chi‐square; *P* = 0.144).

**FIGURE 3:**
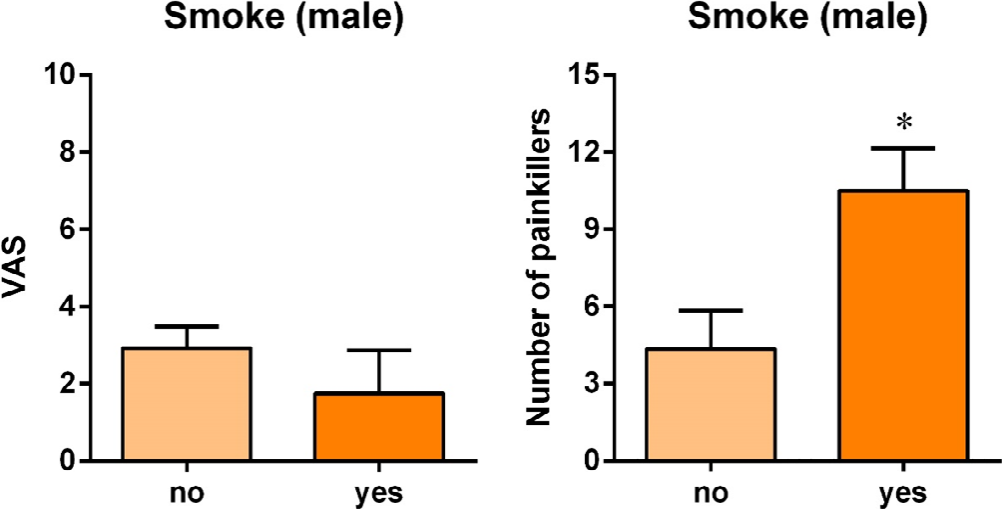
Effect of smoking habit in males on pain score and the number of pain medications taken. *P = 0.057 (Painkillers), Mann–Whitney *U* test. VAS, visual analog scale

When the data were stratified for age (13 in class 1, 15 in class 2 and 20 in class 3) and gender (18 male, 30 female), some relevant differences were found. Considering the smoking factor, male smokers took more pain medications in the first 7 days after surgery than non‐smoking males (*U* = 10.500; *P* = 0.057) (Figure 3).

**FIGURE 4:**
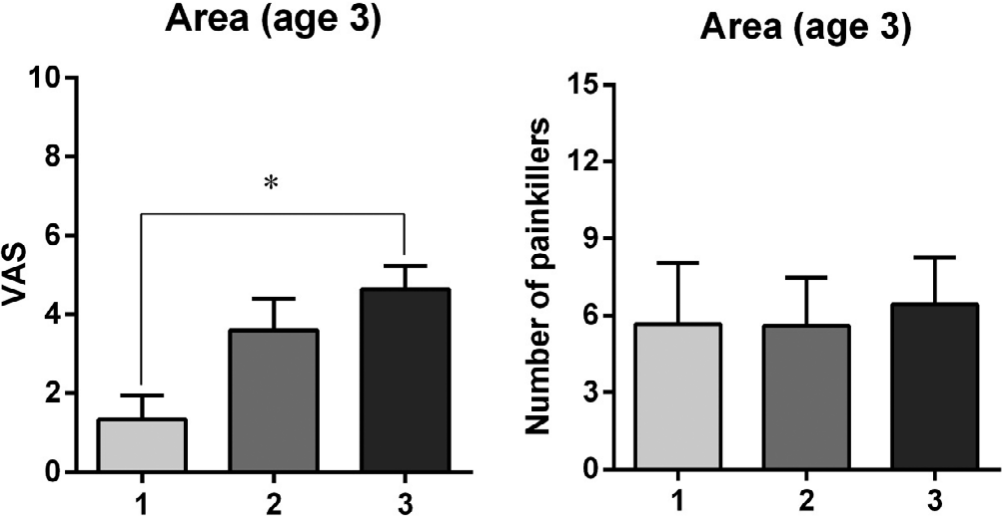
Effect of area of harvested flap in age class 3 on pain score and the number of pain medications taken. *P = 0.022 (1 vs. 3), Kruskal–Wallis test, post‐hoc Dunn's test. VAS, visual analog scale

**FIGURE 5:**
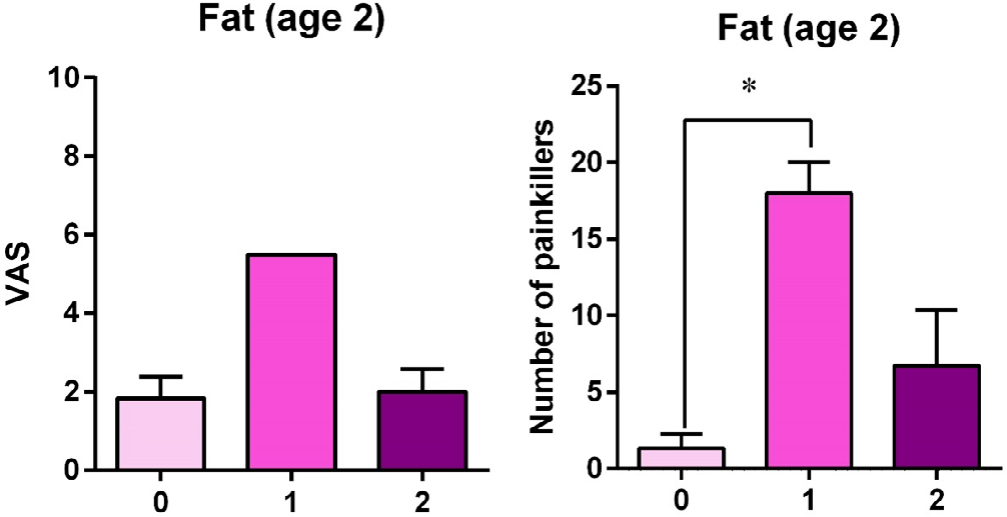
Effect of fat proportion in the harvested flap in age class 2 on pain score and number of pain medications taken. *P = 0.029, Kruskal–Wallis test, post‐hoc Dunn's test. VAS, visual analog Scale

In age class 2, samples with a fat proportion of <50% (category 1 [cat. 1]) were associated with a significantly higher quantity of pain medications (chi‐square = 5.894; *P* = 0.029) (Figure 5) and higher pain scores (chi‐square = 5.403; *P* = 0.067) than samples with no fat (cat. 0). Furthermore, large harvested areas were associated with significantly higher VAS scores in age class 3 than in class 1 (chi‐square = 7.679; *P* = 0.022) (Figure 4).

The logistic binomial regression analysis indicates that use of a bipolar coagulator seemed to have no effect on emergency visit to the clinic (odds ratio = 2.954; *P* = 0.246; 95% CI: 0.474–18.425) or the occurrence of postoperative bleeding (odds ratio = 1.864; *P* = 0.338; 95% CI: 0.521–6.670) (Tables 3 and 4). Stratified data revealed that patients with adipose tissue covering <50% (cat. 1) of in the inner graft surface had a 6.750 times higher probability of making an emergency visit in the first 7 days after surgery (*P* = 0.121; 95% CI: 0.605–75.270), while patients with adipose tissue covering ≥50% (cat. 2) of the inner graft surface had a 4.375 times higher probability of bleeding (*P* = 0.062; 95% CI: 0.928–20.633) than the other groups (Tables 3 and 4). No patient with smoking habit made an emergency visit.

**TABLE 4:** Odds ratios for bleeding

	Wald *t* test	*p*‐value	Odds ratio	95% CI°
Bipolar (yes vs. no)	0.917	0.338	1.864	0.521–6.670
Smoke (yes vs. no)	0.021	0.884	0.875	0.145–5.297
Area	0.078	0.780	1.003	0.984–1.021
Thickness	0.003	0.953	1.026	0.432–2.440
Fat (1 vs. 0)	0.036	0.850	0.833	0.126–5.504
Fat (2 vs. 0)	3.479	0.062	4.375	0.928–20.633

*Note*: Logistic binomial regression test for bipolar, area, and thickness variables on bleeding on global and adipose‐tissue stratified data. °CI, confidence interval.

## 4 DISCUSSION

As the palate is very vascularized, the surgical management of bleeding during CT harvesting is matter of concern. Using a bipolar coagulator could help control bleeding and achieve good mechanical hemostasis; however, even though it is widely used in many surgical fields, to our knowledge, the effect of its use in CT harvesting from the palate has not been described yet. The aim of the present study was to evaluate the implications of using a bipolar coagulator while harvesting CTGs from the palate on the patients' post‐surgical experience in terms of pain and bleeding complications. Emergency visits to the dental clinic were also evaluated because they have important economic implications for the patients.

For decades, CTGs have been harvested using the trapdoor technique (Edel, 1974), and its subsequent modifications have been described by several authors. The rationale for modifying the original FGG harvesting procedure was to reduce postoperative bleeding and pain and to achieve wound closure and healing by first intention. However, it has been recently demonstrated that the thicker the covering flap, the better the outcome (Maino et al., 2018); therefore, in order to obtain a thick covering (primary) flap to prevent postoperative discomfort and pain, we would need to harvest the flap from the deep part of the palatal tissue, but the quality of such a sample would be poor. However, considering that the lamina propria is rich in collagen and the submucosal layer is rich in adipose an glandular tissue (Burkhardt & Hämmerle, 2015), trying to leave a thick residual flap, we would obtain more fatty and glandular tissue instead of white rich‐in‐collagen tissue that leads to better aesthetic outcomes, especially when harvested as a thin sample (Zucchelli et al., 2010; Zucchelli, Stefanini, et al., 2014). Furthermore, considering that studies investigating the difference in postoperative morbidity between connective‐epithelial harvesting (as in the FGG technique) and deep‐tissue harvesting (similar to the trapdoor technique) from the donor site have not shown clear results (Del Pizzo et al., 2002; Wessel & Tatakis, 2008; Zucchelli et al., 2003), in our opinion, the best and current technique is to harvest CT grafts superficially from the palate and de‐epithelize them outside the mouth; hence, this study focused on this CTG harvesting technique.

In our study, the mean VAS value of the maximum pain experienced in the first 7 days was 3.37 (SD, 2.30), which is similar to the values reported by other authors (namely 4.10 (Burkhardt & Hämmerle, 2015), between 3.5 and 4.0 [unclear graphic] (Isler et al., 2018), and 4.47 (Zucchelli, Montebugnoli, et al., 2014), although the mean thickness of the harvested tissues in our study (2.44; SD, 0.75) was higher than that reported in the above studies: 1.83 (SD, 1.03) (Burkhardt & Hämmerle, 2015), 1.51 (SD, 0.15) (Isler et al., 2018), and 2.14 (SD, 0.16) (Zucchelli, Montebugnoli, et al., 2014); this observation of similar maximum VAS values regardless of graft thickness in the three studies is in contrast with the conclusions of each study (Burkhardt & Hämmerle, 2015; Isler et al., 2018; Zucchelli, Mounssif, Mazzotti, Montebugnoli, et al., 2014) but is in accordance with our results.

In our study, the average graft thickness was higher than that reported by the aforementioned studies, as the main purpose of the CTGs in 60% of the cases was to compensate for ridge deficiencies in implant surgery, requiring a graft with a greater thickness than that required for other procedures (Stefanini et al., 2016; Tirone & Salzano, 2018). Interestingly, the mean postoperative pain VAS score in this study was lower than the scores recently reported in studies on sub‐epithelial harvesting from the palate (Amin et al., 2018; Lektemur Alpan & Torumtay, 2020; Maino et al., 2018).

In accordance with the data reported by Burkhardt & Hämmerle, (2015), the present study showed that the dimension of the harvested graft does not affect postoperative discomfort, the self‐reported pain, or the number of analgesic medications taken in the postoperative period. Different findings were reported by Zucchelli et al. (2010), who found a positive relationship between the apico‐coronal height of the donor site and patient‐reported pain.

Taking into consideration the socio‐demographic parameters, the only factor influencing postoperative pain that showed a tendency to significance was smoking, when the data were stratified for gender. Male smokers took more pain medications than non‐smoking males. Similarly, Burkhardt and Hämmerle (2015) showed the influence of smoking on postoperative pain. A reduction in pain tolerance caused by smoking has been widely described in the medical literature (Billert et al., 2007; Smuck et al., 2020). When large‐sized samples were harvested, older patients reported more pain (VAS value) than younger patients.

The mucosal thickness and composition of the palatal tissue vary from patient‐to‐patient and appear to be site specific and gender‐ and age‐dependent (Burkhardt & Hämmerle, 2015). Furthermore, the submucosal layer that is rich in adipose and glandular tissue accounts for 0–79.9% of the thickness of the palatal tissue (Harris, 2003); consequently, the collagen‐rich lamina propria can have a wide variability in thickness. Based on these evidences and the finding that VAS pain after superficial withdrawal with second‐intention healing in our study was lower than the pain reported after deep‐tissue harvesting with first‐intention healing, it could be speculated that the thickness of the harvested sample itself is not related to postoperative discomfort. Our hypothesis was that invasion of the submucosal tissue would be related to a poor outcome or a high probability of postoperative complications. Therefore, we decided to investigate the relationship between the presence of yellow adipose and glandular tissue underneath the sample and postoperative pain and complications. A statistically significant correlation was found between sub‐epithelial tissue invasion and pain, albeit limited to age class two patients; furthermore, patients whose CTG consisted of adipose tissue in the deep layer had a six times higher probability of making an emergency visit during the first postoperative week and a four times higher probability of experiencing postoperative bleeding than those whose CTG had no adipose tissue. Considering these results can be speculated that the superficial tissue harvesting technique with second‐intention healing should be recommended in order to reduce postoperative pain and complications; particularly, the submucosal layer should be invaded as less as possible.

Palatal coverage via different kind of hemostatic treatments has been demonstrated to be beneficial in reducing postoperative pain, (Tavelli et al., 2018, 2019) as a consequence in this study all the sites were protected at the end of surgery with a collagen sponge stabilized by x sutures; the rationale in using the bipolar coagulator is obtaining an immediate thermic hemostasis along surgery in order to simplify the surgical procedures and to speed up the surgery and the sutures.

The use of a bipolar coagulator during surgery was not correlated with the self‐reported VAS score or the number of pain medications used. This means that if an oral surgeon uses this tool during CT harvesting from the palate to improve hemostasis, the healing outcome with regard to pain will not be worsened. A limitation of this study was, as mentioned above, to investigate only the FGG harvesting technique; anyway considering that recently Tavelli et al. (2020) demonstrated in a cadaver split mouth study that the vascular injury, and consequently the bleeding, is worst in trap door harvesting technique when compare with FGG one, can be speculated that the use of the bipolar coagulator could be even more useful while dealing with the former.

## 5 CONCLUSIONS

Bipolar coagulator can be used during CT harvesting from the palate to control bleeding without influencing postoperative pain, bleeding, or emergency visits.

## CONFLICT OF INTEREST

The authors hereby declare that they have no conflict of interest.

## Data Availability

The data that support the findings of this study are available from the corresponding author upon reasonable request.
